# Biochemical Response of the Endogeic Earthworm (*Balanteodrilus extremus*) Exposed to Tropical Soils

**DOI:** 10.1007/s00128-024-03860-7

**Published:** 2024-02-14

**Authors:** E. Lucero Sánchez-del Cid, Jaime Rendón-von Osten, Ricardo Dzul-Caamal, Ma. del Carmen Ángeles González-Chávez, Arturo Torres-Dosal, Esperanza Huerta-Lwanga

**Affiliations:** 1 Agroecología, El Colegio de la Frontera Sur, Unidad Campeche, Av. Polígono s/n, Cd. Industrial, Lerma, Campeche México; 2 Instituto EPOMEX, Universidad Autónoma de Campeche, Campus VI, Av. Héroe de Nacozari 480, Campeche, 24070 México; 3 Programa de Edafología, Colegio de Postgraduados en Ciencias Agrícolas, Montecillo estado de México, Carretera México-Texcoco km 36.5, Montecillo, 56264 México; 4 Salud, El Colegio de la Frontera Sur, Unidad San Cristóbal, San Cristóbal de Las Casas, Chiapas, México; 5 Soil Physics and Land Management, Department of Environmental Sciences, Wageningen University & Research, Droevendaalsesteeg 4, Wageningen, 6708 PB The Netherlands

**Keywords:** Bioindicator, Biomarkers, Agricultural Soils, Mixture of Pesticide Residues

## Abstract

This work evaluated the biochemical responses of the endogeic earthworm *Balanteodrilus extremus* exposed for 14 and 48 days (d) to soils collected from two tropical agricultural systems: maize-sorghum (MS) and soybean-sorghum (SS). A soil without agricultural management (WAM) and the use of pesticides was selected as a reference. The presence of organochlorine (OC) and organophosphate (OP) pesticide residues was quantified in MS and SS soils. Biomarkers of detoxification [glutathione S transferase (GST)], neurotoxicity [acetylcholinesterase (AChE)] and oxidative stress [superoxide dismutase (SOD), catalase (CAT) and lipoperoxidation (LPO)] were evaluated in *B. extremus*. The concentration of OP pesticide residues was higher in SS than in MS. Activity of AChE in *B. extremus* exposed to SS soil for 14 d was significantly more inhibited (78%) than in MS soil (68%). *B. extremus* has been shown to be a good bioindicator of contaminated soils in tropical regions.

**Supplementary Information:**

The online version contains supplementary material available at 10.1007/s00128-024-03860-7.

## Introduction

Agricultural expansion and intensification contribute to the contamination of ecosystems mainly due to the excessive use of pesticides to protect crops (Liu et al. 2015). Maize and soybean are among the top five crops in global agricultural production covering an approximate area of 197 and 121.53 million ha, respectively (USDA 2023). Soybean production has been increasing mainly in South American countries (Argentina and Brazil with 16 and 33% of global production, respectively) and North America (USA with 28% of global production), accounting for around 80% of global production (Dreoni et al. 2022). Maize and soybean crops are responsible for approximately 50% of the most highly hazardous pesticide use globally (Dowler 2020).

Earthworms are used as an indicator of the potential impact of chemicals on soil ecosystems (OECD 1984). Pesticide risk assessments include experiments that are designed to quantify the effect of these pollutants on epigeic earthworms such as *Eisenia fetida* (Pelosi et al. 2013). Studies report that the sensitivity of earthworms to pesticides can vary according to the species and ecological category (Jouni et al. 2021). Endogeic tropical earthworms are recommended for ecotoxicological studies in tropical regions (Datta et al. 2021) due to their ecological importance in the soil and the contact they have with various contaminants through their burrowing activity (Pelosi et al. 2013). However, there is not enough information about this earthworms’ metabolic capacity in the presence of pesticides in tropical regions (Datta et al. 2021). Earthworm enzyme biomarkers can reflect the actual exposure, toxicity, and ecological impact of soil contaminants (Dhiman and Pant 2022).

In Mexico, as in other tropical areas, endogeic earthworm species dominate both natural and disturbed ecosystems (Fragoso et al. 2016). In this work, the endogeic earthworm *Balanteodrilus extremus* was used as a bioindicator of agricultural soil contamination. Hence, the main objectives of this work were (i) to detect organochlorine (OC) and organophosphate (OP) pesticide residues present in maize-sorghum (MS) and soybean-sorghum (SS) soils in a tropical region, (ii) to evaluate the biochemical response of *B. extremus* exposed to these soils for 14 and 48 days (d) and (iii) analyze the relationship between the mixture of pesticide residues present in the MS and SS soils with the biochemical response in *B. extremus.*

## Materials and Methods

The soils were collected from an agricultural area in the Yucatan Peninsula, Mexico in Chencoh ejido, Hopelchén, Campeche (19° 25 × 17.95” N, 89° 48 × 25.58” W). Twelve agricultural soils (0–30 cm depth) were collected from plots with two cropping systems that share sorghum as a rotation crop: maize-sorghum (MS) and soybean-sorghum (SS). Soil samples were transported to the laboratory, frozen, and stored (− 4 °C) in the dark to avoid microbial action or degradation of organic matter and contaminants. Soil without agricultural management (WAM) was considered as a reference and was collected from an area where there is verbal knowledge of farmers who do not apply pesticides were not applied, located 40 km from Hopelchén (19°23’37.74"N, 89°48’0.63” W). These soils were not considered in the pesticide analysis of this study. The work of Sánchez-del Cid (2017) was taken as a reference for the discussion, where they evaluated the presence of organochlorine pesticide residues in the soils of the secondary vegetation where the reference soil for this study was collected.

Soil texture was determined by particle size analysis according to the Bouyoucos (1962) hydrometer method. The pH was determined according to the method AS-02-NOM-021-RECNAT-2000 (NOM-021-RECNAT-2000 2002). The available phosphorus (P) was determined with the Olsen method for neutral to alkaline soils, in which the P was extracted with a NaHCO_3_ solution and adjusted to a pH of 8.5. For acidic soils, P was extracted with a combination of HCl and NH_4_F according to the Bray P-1 method (Bray and Kurtz 1945). Nitrogen (N) was analyzed by the Kjeldahl method (AOAC 1984), which was based on extracting exchangeable ammonium by equilibrating the soil sample with 2 N KCl and was determined by steam distillation in the presence of MgO. Organic matter (OM) was quantified by the titration method according to Gaudette et al. (1974).

The pesticides were detected in the soils according to the methods described by Lopez-Avila and Benedicto (1998) and Wang et al. (2007). Extraction was done in 55 mL perfluoro alkoxy (PFA) polymer Teflon tubes at a temperature of 110 °C microwave extractor (CEM MARS 5). The samples were purified through a glass column and eluted with 30 mL of hexane, 30 mL of a 1:1 mixture of dichloromethane and hexane, and 30 mL of dichloromethane. Samples were evaporated to dryness and resuspended in 50 µL of hexane to quantify contaminants on a Thermo Scientific™ TRACE 1310 TSQ 8000 EVO Triple Quadrupole GC-MS/MS Chromatograph and a Thermo Scientific AI/AS 1310 Autosampler. The pesticides were analyzed and quantified (Table S1) using a mixture of 19 OCs (EPA CLP Organochlorine Pesticides Mix SS, SUPELCO® 4S7426-U) and eight OPs (Organophosphorus Pesticide Mix A, SUPELCO® 48.391).

Earthworms were collected in the agricultural area of Chencoh, Hopelchén. Subsequently, *B. extremus* earthworms from the soil of the site were taken to the laboratory for reproduction according to the method of Huerta et al. (2008). One adult and two juvenile earthworms were placed in plastic containers per 300 g of WAM soil. Soil moisture was determined and adjusted to 22%. Breeding units were incubated in a polystyrene-lined cabinet to maintain a stable temperature (32.7 °C) with permanent darkness. In the first reproductive cycle, the juvenile worms were separated from the adults. The new earthworm generation (presumably without pesticides) was subsequently used in the bioassay of this research. Earthworms were collected in June 2020 and they remained in reproduction until March 2021.

The toxicological test was performed according to the OECD guidelines (OECD 1984) and recommendations made by Jouni et al. (2021). Healthy and mature worms from the generation that were reproduced in the laboratory with a similar weight (0.35 ± 0.02 g) and size (13–15 cm long and 3–5 mm wide) were randomly selected for exposure to agricultural soils. Two treatments with agricultural soil were established: MS and SS and a reference treatment (WAM), each with six repetitions. For each replicate, five earthworms were exposed in 3 L glass tanks (20.5 cm x 25 cm x 16.5) with 500 g of soil. Bioassays were performed at two durations:14 d and 48 d to measure acute and sub-chronic effects, respectively, and to determine the presence of earthworm cocoons. The temperature and photoperiod were the same as those described in the reproduction part.

The weight of the earthworms was measured at the beginning and end of this experiment, and mortality at the end. Earthworm mortality (%) and % body weight change were determined according to Dzul-Caamal et al. (2020), respectively. The average initial weight (P0) of the five earthworms per agricultural soil and the average weight (Pt) after 14 and 48 d were calculated. The formula used was: % change in body weight = [(Pt-P0) / (P0)] *100. The presence of cocoons and neonate individuals was determined after 48 d of exposure in the soil. Subsequently, the earthworms were rinsed with distilled water and stored in Petri dishes on moist filter paper for 24 h (in the dark at 32.7 °C) to void the gut contents.

The earthworms were dissected, and the head (first three segments) was separated from the rest of the body. Separately, samples were homogenized for 20 s in 1 mL of cold 0.1 M phosphate-buffered saline (PBS, pH 7.2), using a PRO250 homogenizer (Pro Scientific®). An aliquot of the homogenate (crude fraction) of the head and body was transferred to a microtube for the determination of LPO content. The remaining homogenate was centrifuged (12,000 g) for 20 min at 4 °C, using a Centrifuge 5417 R (Eppendorf ®) to produce the post-mitochondrial fraction (S9). Aliquots of S9 were stored at − 80 °C until the determination of enzyme activities. Acetylcholinesterase (AChE, EC 3.1.1.7) activity was measured using acetylcholine (ATC) as a substrate according to the colorimetric method of Ellman et al. (1961) with some microplate modifications proposed by Rendón-von Osten et al. (2005). Enzymatic activity was determined kinetically at 414 nm for 5 min. AChE activities were expressed as nmol Acetylcholine hydrolyzed per min mg^− 1^ protein using the molar extinction coefficient (13.6 mM-^1^ cm-^1^). Glutathione S-transferase (GST, EC 2.5.1.18) activity was evaluated following the method of Habig et al. (1974), adapted to a microplate using the conjugation of GSH with 1-chloro-2,4-dinitrobenzene (CDNB; ε = 9.6 mM^− 1^ cm^− 1^). Absorbance was recorded at 340 nm (25 °C) for 3 min kinetics and expressed as nmol CDNB min^− 1^ mg-^1^ protein. Superoxide dismutase (SOD, EC 1.15.1.1) activity was determined using the method described by Misra and Fridovich (1972). 200 µL of body supernatant and 100 µL of 20mM H_2_O_2_ reaction solution contained in an isolation buffer (0.3 M sucrose, 1mM EDTA, 5mM HEPES, and 5mM KH_2_PO_4_) were added to a microplate. Catalase activity (CAT, EC 1.11.1.6) was determined by the method described by Radi et al. (1991) and estimated by the dismutation of hydrogen peroxide (H_2_O_2_) at 240 nm after 0 and 60 s. CAT activity was calculated as mmol of H_2_O_2_ consumed per min mg^− 1^ of protein, using the molar extinction coefficient of 0.043 Mm^− 1^ cm^− 1^. The lipid peroxidation breakdown product (LPO) was determined by the formation of thiobarbituric acid reactive substances (TBARS) according to the method of Buege and Aust (1978) with microplate modifications. LPO results were expressed as nmol TBARS mg^− 1^ total protein, using the molar extinction coefficient (MOC) of 1.56 × 10 5 M^− 1^ cm^− 1^. In all determinations, a water blank was used as a reference. Protein concentration was determined using a protein assay based on the protein-dye binding protocol of Buege and Aust (1978) adapted to a microplate, using bovine γ-globulin as the standard. All these spectrophotometric methods were performed using a multimode reader (Multiskan Spectrum, Thermo Scientific).

The normal distribution of all data was verified; the Kolmogorov-Smirnov test was performed and the Levene test was performed to confirm homoscedasticity in the variances. The biomarkers activity by exposure period was compared between the soils of the agricultural systems and WAM (rank ANOVA, Dunn post hoc test). The average and maximum concentration of each OC and OP pesticide residue, the number of compounds, and the frequency of detection in each agricultural soil systems were calculated. Canonical correspondence analysis was implemented to study the effect of pesticide residues on biomarker response. Spearman’s rho correlation coefficient to calculate the linear correlation between pesticides and biomarkers was used. All data analyses were performed using the statistical software R version 4.2.2 (R Core Team 2022). The R package ggplot2 was used for data presentation.

## Results

The physical and chemical characteristics of agricultural and WAM soils can be seen in Table S2. The soil texture was sandy clay loam in MS and WAM, while that in SS was clay loam. Pesticide residues were detected in the two agricultural systems. There were 25, 19 OCs and seven OPs (Table S3). In the two agricultural systems, endrin ketone (217.48 ± 450.60 ng g^− 1^), dieldrin (80.34 ± 132.52 ng g^− 1^), and endosulfan II (0.30 ± 0.55 ng g^− 1^) pesticide residues were detected with higher frequency and concentration than the other OCs. In the case of OPs, methyl (0.04 ± 0.10 ng g^− 1^), fenchlorphos (0.01 ± 0.02 ng g^− 1^) and disulfoton (7.54 ± 32.85 ng g^− 1^) were detected with high frequency in the study. The concentration of OPs in the SS soils (11.67 ± 14.77 ng g^− 1^) was higher than in the MS soils (4.26 ± 6.93 ng g^− 1^, z = 2.86, *p* < 0.001). In MS, 67% of the samples had more than 19 residues and 33% between 10 and 12, while for SS 40% had more than 19 residues, 30% between 16 and 18, 20% between 13 and 15, and 10% between 10 and 12 (Fig. S1b). In the case of WAM soils, the presence of OC pesticide residues was reported at a lower detection frequency than that of agricultural soils (Table S3).

**Fig. 1:**
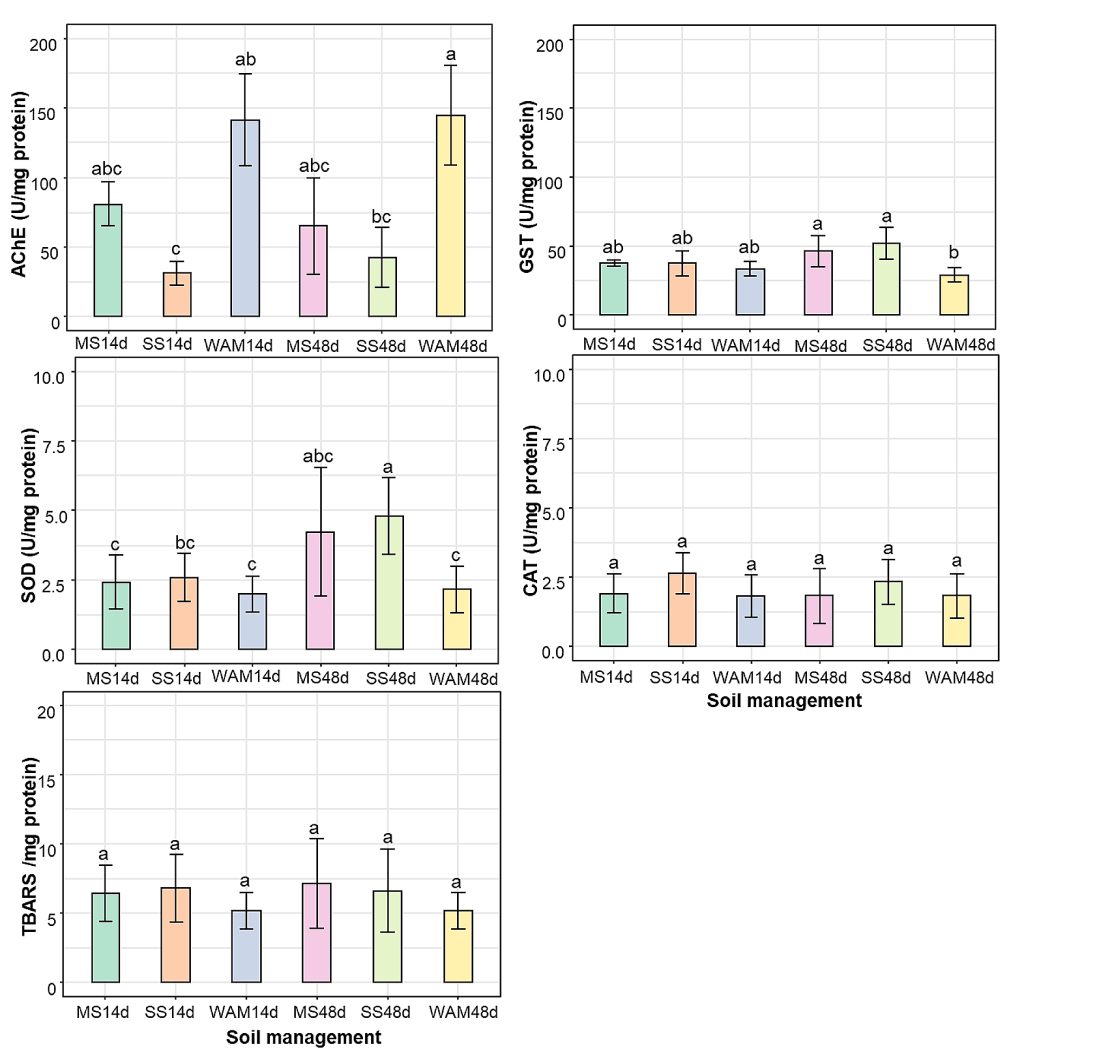
Biochemical response in *Balanteodrilus extremus* earthworms exposed to two agricultural soils and WAM soils; **(a)** enzymatic activity of acetylcholinesterase (AChE); **(b)** Glutathione S-Transferase (GST) activity; antioxidant enzymes activity; **(c)** Catalase (CAT); **(d)** superoxide dismutase (SOD) and **(e)** lipoperoxidation (LPO) concentration (expressed as TBARS). The results are expressed as the mean ± SD (*n* = 15). Different letters indicate significant differences in the soils of the three management systems in the two exposure periods (*p* < 0.05). MS = corn-sorghum; SS = soybean-sorghum; WAM = without agricultural management

Regarding the morphological and physiological changes, it was found that, at 14 and 48 d of exposure, the weight of *B. extremus* decreased in MS and SS soils compared to the WAM reference soils (Fig. S2). The agricultural systems did not have differences between them in weight loss (Fig. S2). However, the mortality of *B. extremus* increased after 48 d of exposure in MS soils and decreased in SS soils (Table S4). No cocoons were found in the WAM soils after 48 d of exposure or in the two agricultural soils; however, neonatal individuals were detected, and those were distributed as follows: MS: 1.6 ± 1.5 and SS: 0.66 ± 0.57 (Table S3). Juvenile individuals were not detected in the WAM soils (Table S4).

In SS soils, AChE activity in *B. extremus* was significantly more inhibited (78%, F = 14.31, *p* < 0.01) than in MS soils (68%) at 14 days of exposure, while at 48 days there were no differences. The AChE activity in SS soils was different from that of the WAM treatment at 14 d (*p* < 0.01) and 48 d (*p* < 0.001), while in MS soils there were no significant differences (Fig. 1a). The activity of GST in *B. extremus* did not vary between the agricultural systems and about the WAM treatment, only at 48 d were significant differences observed in the SS soils ((F = 5.29, p = < 0.001, Fig. 1b). The SOD activity in *B. extremus* was not different between the soils of the two agricultural systems and concerning WAM soils, only after 48 days of exposure were significant differences observed in SS soils (F = 6.88, *p* < 0.01, Fig. 1c). After 48 days of exposure to SS soils, SOD activity in *B. extremus* was higher than after 14 days (*p* < 0.01). Contrarily, the CAT activity in *B. extremus* exposed to MS and SS soils was not different from that of the WAM treatment and neither when comparing between 14 and 48 days (F = 2.25, *p* > 0.05, Fig. 1d). Similarly, the LPO levels in *B. extremus* neither showed no significant differences between any of the treatments (Fig. 1e).

**Fig. 2:**
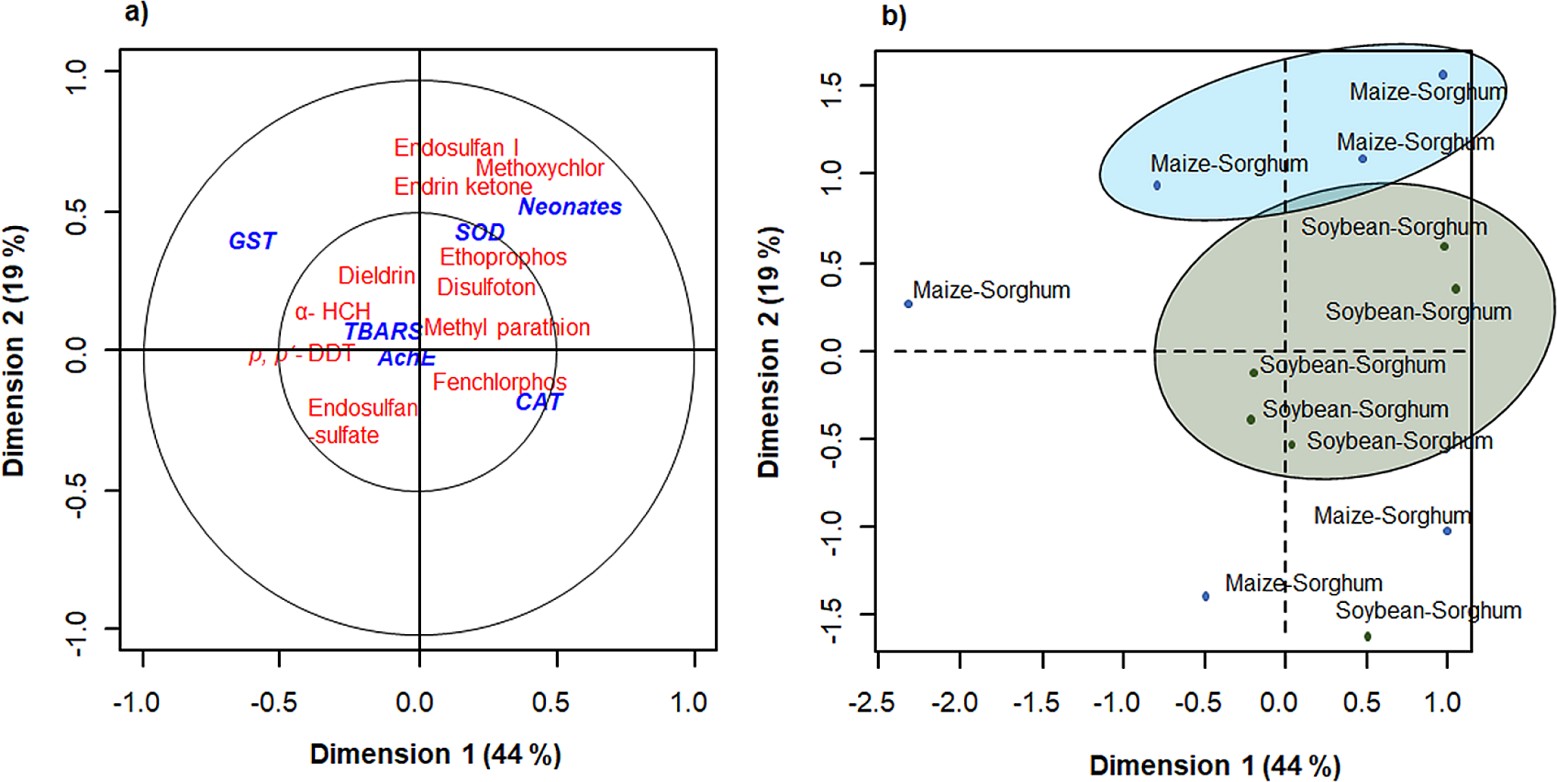
Canonical correspondence analysis of physiological and biochemical response by *Balanteodrilus extremus***(a)** regarding organochlorine and organophosphate pesticide residues concentration and **(b)** by soils of agricultural systems in the ejido of Chencoh, Hopelechén, Campeche

The canonical correlation analysis explained 44% of the variance in dimension 1 and 19% in dimension 2 (Fig. 2) and clearly showed the correlation between the response of the biomarkers at 48 d of exposure and the presence of pesticides. Ethoprofos, methyl parathion, and disulfoton present in MS and SS soils correlated with oxidative stress (SOD) of *B. extremus* and negatively with increased AChE activity. Spearman’s correlation analysis confirmed, as shown in Fig. 2, that ethoprophos (rho = 0.75, *p* ≤ 0.01) and methoxychlor (rho = 0.59, *p* ≤ 0.05) were correlated positively with the presence of neonatal individuals of *B. extremus.*

## Discussion

AChE activity in *B. extremus* exposed to agricultural soils varied according to the agricultural system (Fig. 1a). The SS soils were those that inhibited AChE activity concerning the WAM treatment, while no differences were observed in the MS soils.The higher concentration of OP in SS could cause AChE to be inhibited in SS soils at 14 and 48 d of exposure (Table S3). Koelle (2013) mentioned that AChE is an enzyme that is involved in breaking down the neurotransmitter acetylcholine into choline and acetic acid. The pesticides OPs and carbamates are the main ones that impair this activity (Dhiman and Pant 2022). The percentage of inhibition in this study (78%) with respect to soils of the WAM treatment was higher than that of Andrade-Herrera et al. (2019) reported in agricultural soils of Quintana Roo, Mexico for *E. fetida* (50%) and lower (84–97%) than Sanchez-Hernandez et al. (2014) observed for A*porrectodea caliginosa.* The AChE activity at 48 d was not different in relation to that at 14 d (Fig. 1a) although it was different from the WAM treatment, which indicated that the activity did not recover within a period of 34 d.

Panda and Sahu (2004) showed that the maximum inhibition (46%) of *Drawida willsi* (Oligochaeta) was at 9 d and fully recovered within 45 and 75 d after malathion (2.2 mg kg ^1^) and carbofuran (1.1 mg kg ^1^), respectively. In the present study, *B. extremus* was exposed to more than one compound at concentrations lower than those used by the authors. The most common OP mixture in SS soils was fenchlorphos (0.03 ng g^− 1^) + methyl parathion (0.10 ng g^− 1^) + disulfoton (34.04 ng g^− 1^).

The exposure of *B. extremus* to agricultural soils for 14 d did not significantly induce GST activity compared to that of the WAM treatment but it did at 48 d. Differences in GST activation are observed in the literature and seem to depend on the contaminant, the dose or concentration and the species of earthworm used (Jouni et al. 2021). For example, Schreck et al. (2008) found that in short periods (3 d) of exposure to chlorpyrifos, the activity of GST in *A. caliginosa* increased, but in the long term, the activity decreased. Yao et al. (2020) reported that GST activity in *E. fetida* remained low in groups exposed to high levels of tifluzamide (1.0 and 10.0 mg kg ^1^) in short-term exposure periods. Jouni et al. (2021) analyzed the effect of chlorpyrifos and ethyl paraoxon (1 and 1.3 mg kg ^1^) on *Aporrectodea chlorotica* and *A. caliginosa* and reported that after 7 d of exposure, GST activity did not increase in either species. In our study, exposure at 14 d did not induce differences in terms of the activity of the WAM treatment; however, it cannot be excluded that it occurred in the first days of exposure.

The SOD enzyme is the first line of defense in the antioxidant system and tends to increase in the first days of exposure (Gu et al. 2021). Nevertheless, the results of this study showed that in SS soils at 14 d, the SOD activity in *B. extremus* was not different from the WAM treatment, but it was different at 48 d of exposure. Zhang et al. (2020) found that SOD activity in *E. fetida* exposed to the insecticide sulfoxaflor had a dose-response relationship. The authors noted that at concentrations of 0.1 mg kg ^− 1^, the activity was not different from the control at any exposure time (2, 7, 14, 28, 42, and 56 d); while at 0.5 and 1 mg kg ^1^ it was at 42 d. Avoidance behavior in earthworms exposed to pesticides is well-known in the literature (Yu et al. 2019). Therefore, a hypothesis could be that the earthworms exposed for 14 d avoided consuming the soil. This would explain why SOD activity was not significantly increased or inhibited in the agricultural soils when compared to WAM treatment.

In this study, CAT activity in *B. extremus* did not differ from WAM treatment in either of the two agricultural systems, nor did it vary between the duration of exposure (Fig. 1d). In this study compared to CAT, SOD had a more sensitive response to agricultural soils at 48 d, which was also reported for *E. fetida* (Zhang et al. 2020). Studies mentioned that TBARS are cytotoxic end products of cellular lipid peroxidation; the content could reflect whether the body is subject to lipid peroxidation (Zhang et al. 2020). In our study, the LPO levels in *B. extremus* exposed to agricultural soils did not show differences with the WAM treatment at 14 or 48 d of exposure. These results are similar to those reported by Zhang et al. (2020) when *E. fetida* was exposed to 0.1 mg kg ^1^ of the insecticide sulfoxaflor; however, in higher concentrations, it was activated at 7 d. As commented for GST, it cannot be excluded that it occurred in the first days of exposure. These results showed that SOD and GST activity at 48 d protected *B. extremus* from lipid peroxidation.

The earthworms exposed to the WAM treatment did not decrease their weight or mortality at 14 and 48 d of exposure. In the soils of both agricultural systems, the weight of *B. extremus* decreased and mortality was observed. This could be because earthworms, when exposed to pesticides, regulate the intake of food, and decrease the rate of consumption, which affects the growth rate and results in weight loss (Usmani et al. 2020). Mortality varied by treatment and tended to increase after 48 d of exposure in MS soils. Survival experiments under chronic exposure indicated that the mortality of adult earthworms increases in the presence of a mixture of various active substances (Basley and Goulson 2017).

Earthworms in these areas are exposed to the interaction of different active substances and their degradation products. Yasmin and D’Souza (2007) mentioned that after removing pesticide-treated soils, earthworms take between 4 and 8 weeks to recover to their normal growth and reproduction rates. However, in the field, the continuous exposure of earthworms to pesticides can have consequences from generation to generation if the compounds are persistent in the soil or if there is a transmission of some harmful effects from parents to offspring. In the case of this study, exposure to OC pesticides in high concentrations such as endrin ketone and dieldrin could have an intergenerational effect on *B. extremus.*

Similar OC pesticide residues as the ones found in this study have been reported in agricultural soils (maize, wheat, cotton, and citrus crops) from Sonora, México (Leal et al. 2014). The authors detected the residues endrin, dieldrin, endosulfan, and DDT in higher concentrations compared to other OCs such as lindane and methoxychlor, similar to what is reported in this work (Table S3). The concentration of dieldrin in a soil sample in SS exceeded (> 500 ng g^− 1^) the Target Dutch and Internvention Values (2000) soil quality standards (500 ng g^− 1^); as well as endrin ketone (40 ng g^− 1^) in 38% of the SS samples (72 to 757 ng g^− 1^) and in 67% of the MS samples (46 to 2529 ng g^− 1^). The results showed that the SS soils had higher OP concentrations than those of MS, this could be due to the difference in the type of soil texture. The clay loam texture in SS soils is characterized by a higher percentage of clay than the sandy clay loam. Regarding this, Copaja and Gatica-Jeria (2021) mentioned that in soils with a high percentage of clay, the absorption of OP compounds increases and therefore, they are retained longer in the soil. The rate and frequency of pesticide application are also parameters that could influence the difference between concentrations (Bedos et al., 2002) and should be considered in future studies.

Although disulfoton and methyl parathion were detected with higher concentrations compared to the rest of the OPs, ethoprofos that correlated with SOD was among the OPs with the lowest concentrations in the study (0.01 ng g^− 1^). Yang et al. (2018) observed that mixtures of two and four pesticides exhibited synergistic effects on *E. fetida*, but also in another combination they showed antagonistic effects. Another important result was the presence of neonates at 48 d of exposure and its significant correlation with ethoprophos (rho = 0.75, *p* ≤ 0.01) and methoxychlor. (rho = 0.59, *p* ≤ 0.05). These two pesticides are classified as endocrine disruptors (Arena et al. 2018). However, there is no clear information on the effect of these pesticides on earthworm reproduction, and the effects of mixing pesticides are less clear.

The results of this article offer a better understanding of the impact of the mixture of pesticide residues to which earthworms are exposed in agricultural soils. *Balanteodrilus extremus* is certainly a suitable bioindicator of contamination in tropical soils. Earthworms play an important role in agricultural soils, therefore, it is important to consider management practices that reduce the need for pesticide use and promote better conditions for earthworms. These results may also support the need to consider remediation measures for soils contaminated by pesticides of historical use such as endrin ketone and dieldrin.

## Conclusion

Endrin ketone, dieldrin, and endosulfan II were detected in higher concentrations than the other compounds in 83 to 100% of the soil samples. Ethoprophos, disulfoton, and methyl parathion together with methoxychlor, endosulfan I, and endrin ketone were grouped with SOD activity. This indicates that both OP and OC pesticide residues influenced the oxidative response of *B. extremus*. The SS soils were shown to have a greater negative impact than the MS soils on AChE activity in *B. extremus* at 14 d of exposure and in relation to the WAM treatment, in the two exposure periods. This work highlights the impact mainly of SS agricultural soils on the biochemical response of *B. extremus*, an endogenous earthworm that is present in agroecosystems and that could be a good bioindicator of soil contamination in tropical environments.

### Electronic supplementary material

Supplementary table for Tables (PDF 38 kb)
