# Supplementary material for: Biochemical Response of the Endogeic Earthworm (Balanteodrilus extremus) Exposed to Tropical Soils
Source: Bull Environ Contam Toxicol. 2024 Feb 14;112(2):35. doi: 10.1007/s00128-024-03860-7 (PMC10867048; doi:10.1007/s00128-024-03860-7)
Supplement: Supplementary file 1 — Supplementary table for Tables (PDF 38 kb) [file 128_2024_3860_MOESM1_ESM.docx]

**Biochemical response of the endogeic earthworm** **(*Balanteodrilus extremus*) exposed to tropical soils**

E. Lucero Sánchez-del Cid^1^, Jaime Rendón-von Osten^2^, Ricardo Dzul-Caamal^2^, Ma. del Carmen Ángeles González-Chávez^3^, Arturo Torres-Dosal^4^, Esperanza Huerta-Lwanga^1,5^

**Table S1.** Organochlorine (OC) and organophosphate (OP) pesticides analyzed in in soils collected from Maize-Sorghum and Soybean-Sorghum cropping systems. LOD: limit of detection.

| **Compound** | **Abbreviation**  **by compound** | **Abbreviation by**  **group** | **LOD (ng g^-1^)** |
| --- | --- | --- | --- |
| Alpha-Hexachlorocyclohexane | α-HCH | HCHs | 4.24 |
| Beta- Hexachlorocyclohexane | β-HCH |  | 5.86 |
| Delta- Hexachlorocyclohexane | δ-HCH |  | 2.82 |
| Gamma- Hexachlorocyclohexane | γ-HCH |  | 5.13 |
|  |  |  |  |
| Endrin |  | DRINEs | 13.40 |
| Endrin ketone |  |  | 7.29 |
| Dieldrin |  |  | 5.46 |
| Aldrin |  |  | 5.39 |
|  |  |  |  |
| Endosulfan I |  | ENDOs | 6.59 |
| Endosulfan II |  |  | 7.83 |
| Endosulfan sulfate |  |  | 6.75 |
|  |  |  |  |
| Dichlorodiphenyltrichloroethane | *p, p´-*DDT | DDTs | 8.55 |
| Dichlorodiphenyldichloroethylene | *p, p´-*DDE |  | 5.39 |
| Dichlorodiphenyldichloroethane | *p, p´-*DDD |  | 6.34 |
|  |  |  |  |
| Heptachlor |  | HEPTCHLs | 5.39 |
| Heptachlor epoxide |  |  | 6.86 |
|  |  |  |  |
| Trans chlordane |  |  | 5.72 |
| Cis chlordane |  |  | 5.99 |
| Methoxychlor |  |  | 9.46 |
|  |  |  |  |
| Chlorpyrifos |  | OPs | 1.08 |
| Disulfoton |  |  | 0.2 |
| Azinphos-methyl |  |  | 1.08 |
| Ethoprophos |  |  | 0.4 |
| Fenchlorphos |  |  | 1.08 |
| Parathion-methyl |  |  | 2.43 |
| Prothiophos |  |  | 0.08 |
| Diclorvos |  |  | 0.2 |

**Table S2.** Characterization of soils by agricultural management and description of physical and chemical characteristics. Mean and standard deviation (±SD) per parameter. OM: organic matter, P: phosphate, N: nitrogen, CL: clay loam, SCL: sandy clay loam. Different bold letters represent significant differences between soils from different soil management systems.

| **Soil samples** | **Soil**  **Systems** | **OM**  **%** | **pH** | **N**  **mg/kg** | **P**  **mg/kg** | **Sand**  **%** | **Clay**  **%** | **Silt**  **%** | **Texture** |
| --- | --- | --- | --- | --- | --- | --- | --- | --- | --- |
| 1 | MS | 3.8 | 8.2 | 21.0 | 16 | 51.7 | 25.9 | 22.3 | SCL |
| 2 |  | 3.4 | 7.2 | 24.0 | 7.0 | 50.4 | 23.9 | 26.0 | SCL |
| 3 |  | 6.2 | 7.5 | 17.5 | 5.5 | 46.0 | 27.2 | 26.3 | SCL |
| 4 |  | 3.9 | 7.6 | 19 | 13.1 | 48.7 | 25.0 | 26.1 | SCL |
| 5 |  | 3.8 | 7.2 | 24.5 | 14.91 | 57.1 | 26.8 | 28.7 | SCL |
| 6 |  | 3.8 | 6.3 | 28 | 45.3 | 49.7 | 28.8 | 21.4 | SCL |
|  |  | 4.1±1.01**a** | 7.3±0.6**a** | 22.3±3.8**a** | 16.9±14**a** | 50.6±3.7**ab** | 26.2±1.7**a** | 25.1±2.7**a** |  |
|  |  |  |  |  |  |  |  |  |  |
| 7 | SS | 5.9 | 6.8 | 26.3 | 11.83 | 48.9 | 29.9 | 21.0 | SCL |
| 8 |  | 6.5 | 8.0 | 28 | 8.48 | 50.0 | 27.9 | 22.0 | SCL |
| 9 |  | 2.4 | 7.0 | 24.5 | 3.22 | 31.0 | 39.6 | 26.3 | CL |
| 10 |  | 1.7 | 7.0 | 21 | 8.07 | 54.7 | 21.0 | 24.1 | SCL |
| 11 |  | 4.1 | 6.0 | 24.5 | 9.89 | 42.9 | 31.9 | 25.0 | CL |
| 12 |  | 4.0 | 6.5 | 19.3 | 1.4 | 35.7 | 36.5 | 27.7 | CL |
|  |  | 4.1±1.8**a** | 6.8±0.6**a** | 23.9±3.2**a** | 7.1±4.0**a** | 43.8±9.0**b** | 31.1±6.5**a** | 24.3±2.5**a** |  |
|  |  |  |  |  |  |  |  |  |  |
| 1 | WAM | 6.5 | 7.2 | 21.0 | 10.5 | 63.12 | 20.88 | 16.0 | SCL |
| 2 |  | 4.6 | 7.8 | 15.8 | 4.9 | 57.12 | 24.88 | 18.0 | SCL |
| 3 |  | 3.7 | 8.1 | 26.3 | 4.3 | 50.68 | 30.88 | 18.4 | SCL |
|  |  | 4.9±1.7**a** | 7.7±0.4**a** | 21.5±1.7**a** | 6.6±3.3**a** | 56±6.2**a** | 25.5±5.0**a** | 17.4±1.3**b** |  |

**Table S****3.** Concentration of pesticide residues (ng g^-1^) detected in soils collected from Maize-Sorghum (MS) and Soybean-Sorghum (SS) cropping system and soils without agricultural management (WAM) in the ejido of Chencoh, Hopelechén, Campeche. ND: not detected; NA: not applicable; NAN: not analyzed SD: standard deviation.

|  |  |  | **MS** |  |  |  | **SS** |  |  |  |  |  | **WAM*** |  |
| --- | --- | --- | --- | --- | --- | --- | --- | --- | --- | --- | --- | --- | --- | --- |
| General  frequency  (freq %) | Compound  (classification) | % freq | Maximum | Mean |  | % freq | Maximum | Mean |  | Total  Mean±SD |  | % freq | Maximum | Mean |
| 84-100 | Dieldrin | 100 | 472 | 122 |  | 100 | 505 | 116 |  | 80.34±132 |  | 16 | 0.49 | NA |
|  | Endosulfan II | 83 | 2.55 | 0.82 |  | 83 | 1.70 | 0.38 |  | 0.30±0.55 |  | ND | NA | NA |
|  | Fenchlorphos | 83 | 0.07 | 0.03 |  | 100 | 0.08 | 0.03 |  | 0.01±0.02 |  | ND | NA | NA |
|  | Trans chlordane | 100 | 0.23 | 0.17 |  | 83 | 0.26 | 0.10 |  | 0.08±0.08 |  | NAN | NA | NA |
|  | α-HCH | 100 | 0.06 | 0.03 |  | 66 | 0.06 | 0.01 |  | 0.02±0.02 |  | ND | NA | NA |
|  | β-HCH | 83 | 0.98 | 0.09 |  | 83 | 0.17 | 0.05 |  | 0.07±0.16 |  | 16 | 0.40 | NA |
|  | Cis-chlordane | 100 | 0.44 | 0.19 |  | 66 | 0.33 | 0.15 |  | 0.03±0.10 |  | ND | NA | NA |
|  | Endosulfan I | 83 | 0.67 | 0.27 |  | 83 | 1.70 | 0.27 |  | 0.18±0.22 |  | ND | 0.30 | NA |
| 76-83 | E. sulfate | 100 | 0.26 | 0.09 |  | 66 | 0.26 | 0.08 |  | 0.06±0.07 |  | ND | NA | NA |
|  | Endrin ketone | 100 | 2529 | 364 |  | 66 | 757 | 110 |  | 217±450 |  | ND | NA | NA |
|  | Methyl parathion | 83 | 0.28 | 0.12 |  | 83 | 0.76 | 0.14 |  | 0.04±0.10 |  | NAN | NA | NA |
|  | *p, p´-*DDT | 83 | 0.64 | 0.29 |  | 83 | 0.30 | 0.13 |  | 0.15±0.19 |  | ND | NA | NA |
|  | Disulfoton | 83 | 24.23 | 4.88 |  | 66 | 196 | 34.06 |  | 7.54±32.85 |  | NAN | NA | NA |
|  | Heptachlor epoxide | 83 | 0.21 | 0.10 |  | 66 | 0.26 | 0.09 |  | 0.05±0.07 |  | 16 | 0.38 | NA |
|  | Methoxychlor | 83 | 0.26 | 0.11 |  | 66 | 0.26 | 0.10 |  | 0.06±0.09 |  | 16 | 1.02 | NA |
| 51-75 | δ-HCH | 66 | 0.23 | 0.05 |  | 66 | 0.12 | 0.05 |  | 0.02±0.05 |  | 16 | 0.18 | NA |
|  | γ-HCH | 83 | 1.20 | 0.15 |  | 50 | 0.16 | 0.03 |  | 0.05±0.20 |  | ND | NA | NA |
|  | Azinphos methyl | 83 | 1.41 | 0.51 |  | 33 | 0.69 | 0.27 |  | 0.22±0.36 |  | NAN | NA | NA |
|  | Ethoprophos | 66 | 0.07 | 0.02 |  | 50 | 0.05 | 0.02 |  | 0.01±0.02 |  | NAN | NA | NA |
|  | *p, p*´-DDE | 50 | 0.07 | 0.02 |  | 50 | 0.07 | 0.02 |  | 0.01±0.02 |  | 66 | 0.68 | 0.49±0.14 |
|  | *p, p´-*DDD | 50 | 0.73 | 0.19 |  | 33 | 0.27 | 0.06 |  | 0.04±0.14 |  | ND | NA | NA |
| 16-50 | Prothiofos | 50 | 0.25 | 0.08 |  | 33 | 0.42 | 0.12 |  | 0.04±0.10 |  | NAN | NA | NA |
|  | Chlorpyrifos | 50 | 0.41 | 0.11 |  | 33 | 0.29 | 0.05 |  | 0.03±0.10 |  | NAN | NA | NA |
|  | Heptachlor | 0 | ND | ND |  | 66 | 0.05 | 0.01 |  | 0.003±0.01 |  | ND | NA | NA |
|  | Endrin | 16 | 3.43 | 0.57 |  | 16 | 14.70 | 2.45 |  | 0.57±2.52 |  | ND | NA | NA |
|  |  |  |  |  |  |  |  |  |  |  |  |  |  |  |
| 88 | ΣHCHs | 94 | 2.19 | 0.25**a** |  | 83 | 0.47 | 0.11**a** |  | 0.18±0.36 |  | NA | NA | NA |
| 91 | Σdrines | 94 | 2529 | 403**a** |  | 88 | 758 | 193**a** |  | 298±447 |  | NA | NA | NA |
| 93 | ΣENDOs | 100 | 2.73 | 0.66**a** |  | 94 | 2.16 | 0.44**a** |  | 0.55±0.59 |  | NA | NA | NA |
| 69 | ΣDDTs | 72 | 1.24 | 0.29**a** |  | 66 | 0.52 | 0.12**a** |  | 0.20±0.26 |  | NA | NA | NA |
| 93 | ΣCHLs | 77 | 0.46 | 0.19**a** |  | 77 | 0.51 | 0.18**a** |  | 0.18±0.16 |  | NA | NA | NA |
| 54 | ΣHEPTCHLs | 55 | 0.21 | 0.07**a** |  | 66 | 0.26 | 0.05**a** |  | 0.06±0.07 |  | NA | NA | NA |
| 94 | ΣOPs | 100 | 24.23 | 4.27**a** |  | 88 | 197 | 11.67**b** |  | 7.97±32.83 |  | NA | NA | NA |

* Reference values by Sánchez- del Cid (2017).

**Table S4**. Description of the morphological and physiological parameters in b. extremus at 14 and 48 d of exposure. MS: maize-sorghum; SS: soybean-sorghum; WAM: without agricultural management.

| **Parameter** | **MS** | **SS** | **WAM** |
| --- | --- | --- | --- |
| Mortality % (14 d) | 3 | 10 | 0 |
| Mortality % (48 d) | 20 | 7 | 0 |
| Number of cocoons (48d) | 0 | 0 | 0 |
| Number of neonates (48d) | 1.6±1.5 | 0.66±0.57 | 0 |

**
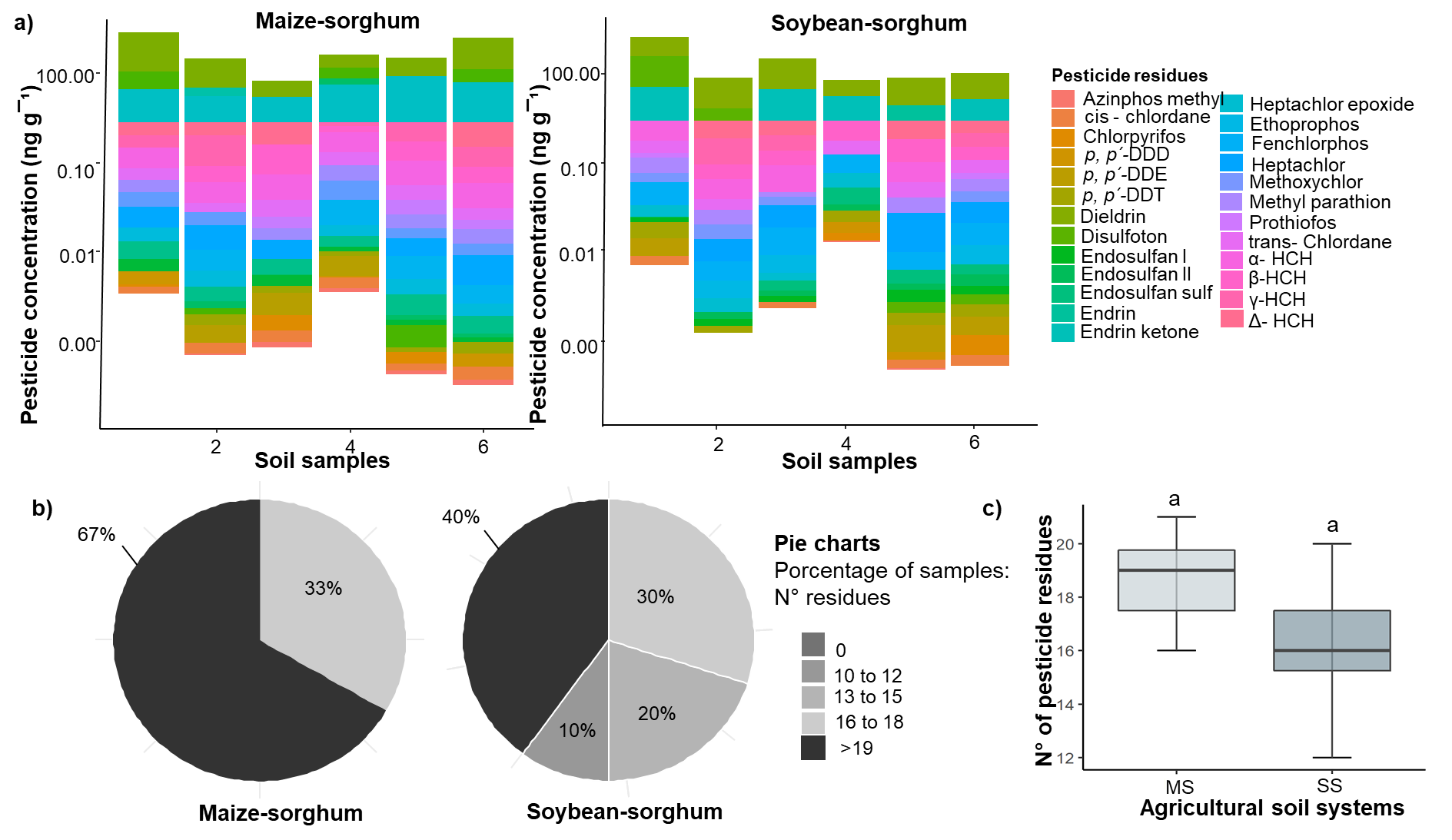
Fig. S1.** Pesticide residues by agricultural system; a) total concentration of residues by soil sample; b) number of pesticides by percentage; c) number of pesticide residues detected by Maize-sorghum (MS) and Soybean-sorghum (SS) system. The number of residues in the soil was divided by ranges and the distribution of each range is presented in the pie charts.

**
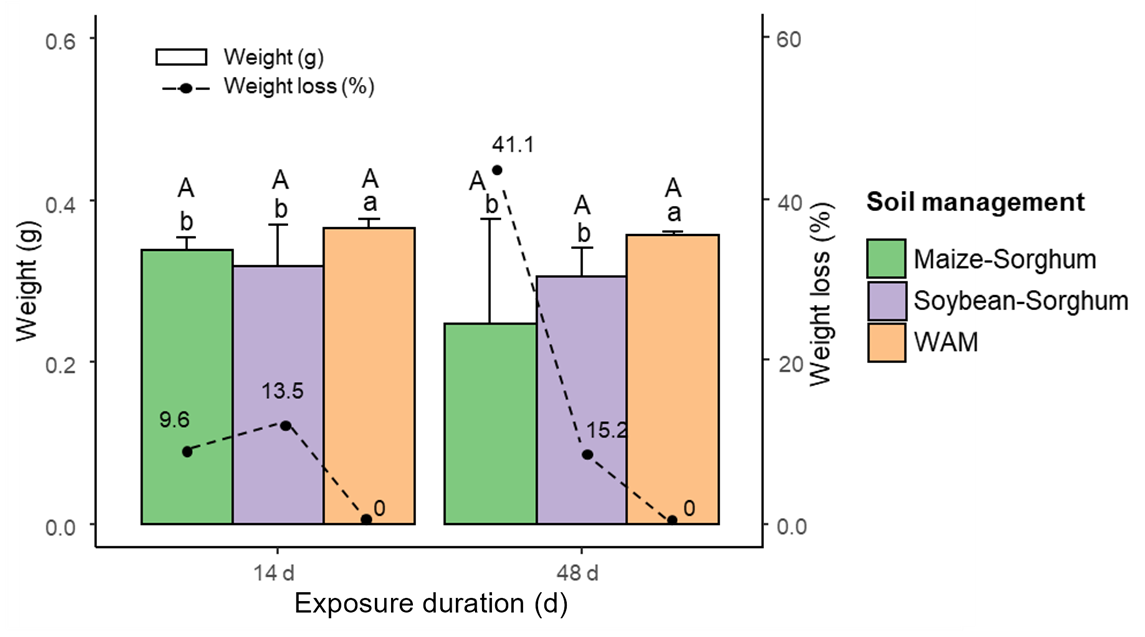
**

**Fig. S2.** Changes in fresh weight of earthworms after 14 and 48 d of exposure. The bars are the mean weight (g) in the different agricultural managements (±SD; n=15). The lines represent the weight loss in percentage. Different capital letters indicate significant difference among the exposure time (14 vs 48 d) in the same agricultural system and lower-case letters indicate significant differences among the soils of the three management systems (p<0.05). WAM= without agricultural management.
